# DUS evaluation of nine intersubgeneric hybrids of *Paeonia lactiflora* and fingerprint analysis of the chemical components in the roots

**DOI:** 10.3389/fchem.2023.1158727

**Published:** 2023-03-10

**Authors:** Shiyi Xu, Weili Liu, Xiubo Liu, Chen Qin, Lianqing He, Panpan Wang, Lingyang Kong, Xi Chen, Zhiyang Liu, Wei Ma

**Affiliations:** ^1^ School of Pharmacy, Heilongjiang University of Chinese Medicine, Harbin, China; ^2^ Experimental Training Center, Heilongjiang University of Chinese Medicine, Harbin, China; ^3^ Jiamusi College, Heilongjiang University of Chinese Medicine, Harbin, China; ^4^ Harbin Academy of Agricultural Sciences, Harbin, China

**Keywords:** *Paeonia lactiflora* pall., DUS, fingerprint analysis, intersubgeneric hybrid, paeoniflorin, stoichiometric analysis, HPLC

## Abstract

Intersubgeneric hybrids of *Paeonia lactiflora* (*Paeonia lactiflora* pall., *P. lactiflora.*) cover a huge variety of systems in the genus *Paeonia*. In recent years, many studies have confirmed that the intersubgeneric hybrids of *P. lactiflora.* are rich in paeoniflorin and other medicinal ingredients, however, it has always proved difficult to clarify the medicinal value of the hybrids and whether they can be used for medicinal purposes. In this study, the consistency of the plant population was evaluated through DUS evaluation, in order to clarify whether the selected research materials had stability and consistency within the population and specificity between populations. The differences between the paeoniflorin contents in the roots of the nine intersubgeneric hybrids of the *P. lactiflora.* varieties and two medicinal varieties were critically compared. The differences in the chemical components of the roots of nine intersubgeneric hybrids of *P. lactiflora.* and reference medicine substances of *P. lactiflora.* and *Paeonia anomala* subsp. *veitchii* (Lynch) D. Y. Hong and K. Y. Pan (*Paeonia veitchii Lynch*., *P. veitchii*.) were explored *via* stoichiometric and chemical fingerprint high performance liquid chromatography analyses. The results showed that there were significant differences in the chemical compositions between the intersubgeneric hybrids of *P. lactiflora*. and the medicinal reference materials, and the contents of paeoniflorin were elevated such that the hybrids could be used as the raw material for extraction of paeoniflorin, thus providing an opportunity to explore the medicinal value of the hybrids. This study explored the key differential components among the varieties and provides a reference and basis for the study of the medicinal value and the identification of the intersubgeneric hybrids of the *P. lactiflora.* varieties.

## 1 Introduction

*P. lactiflora.* is a perennial herb with both ornamental and medicinal value (Ahmad et al., 2018; Yan et al., 2021). The plant resources are widely distributed in temperate Eurasia. *P. lactiflora.* has a long history of cultivation in China and is one of the “six famous flowers” in China (Liu and Cheng, 2020; Li et al., 2021). *P, veitchii Lynch.* (Wei et al., 2022) and *P. lactiflora.* (Sheng et al., 2020) are the original plants of Paeoniae Radix Rubra and Paeoniae Radix Alba which are traditional Chinese medicine (TCM) (Chinese Pharmacopoeia Commission, 2020)*.* In addition to the medicinal value of the roots, the seeds of *P. lactiflora.* can also be used for production of α-linolenic acid (Wu et al., 2020; Yu et al., 2021). However, in recent years, with the continuous reduction of natural resources, artificial cultivation of *P. lactiflora.* has become the main source of TCM. To expand the available resources, researchers have turned their attention to intersubgeneric hybrids of *P. lactiflora.*, and found that the content of paeoniflorin (PA) in the roots of some intersubgeneric hybrids of *P. lactiflora.* meets the medical standards (Sun et al., 2021). However, due to hybridization and domestication, the varieties of the intersubgeneric hybrids of *P. lactiflora.* are complex, and the chemical components are also unstable. Therefore, it is necessary to undertake in-depth studies to determine the chemical differences between the intersubgeneric hybrids of *P. lactiflora.* and the associated medicinal properties of these varieties.

The morphological characterization of *P. lactiflora.* is related to such factors as the environment (Sun et al., 2022), their development and distant hybridization (Zhang et al., 2019). The morphology is not only manifested in flower type, flower color (Tang et al., 2018; Tang et al., 2020; Luo et al., 2021) and plant morphology, but also in the differences in the chemical components (Wang et al., 2014) and the related gene expression (Zhao et al., 2014; Gao et al., 2016). The varieties of *P. lactiflora.* on the international market can be divided into two categories: Chinese *P. lactiflora.* varieties and hybrid *P. lactiflora.* varieties (Yang et al., 2020). The parent of the Chinese *P. lactiflora.* variety group is a single original species, which is *P. lactiflora.*, the offspring bred from it being diploid. The parents of the hybrid *P. lactiflora.* cultivar group come from two or more original species. Their parents are both diploid and tetraploid, so the ploidy of their offspring is diversified, and the diploid, triploid and tetraploid forms will appear (Wu et al., 2021; Cui et al., 2022). The hybrids between subgenus *P. lactiflora.* are bred by hybridizing herbaceous *P. lactiflora.* and woody *Paeonia×suffruticosa*. The intergeneric hybrids varieties are the dominant varieties cultivated through continuous hybridization, so their parents are relatively complex. Therefore, there are rich differences in morphology and chemical composition between different varieties of intergeneric hybrids of *P. lactiflora*. The intergeneric hybrids possess certain advantages with respect to stress resistance and ornamental suitability.

The Chinese Pharmacopoeia (Ch.P) (Chinese Pharmacopoeia Commission, 2020) stipulates that PA (Xiang et al., 2020) is a marker compound for the identification of the quality of Paeoniae Radix Rubra and Paeoniae Radix Alba*.* However, although PA can be used to confirm the quality of medicinal materials, it cannot serve as the only indicator for identification of varieties and sources. In recent years, chemical fingerprint analysis (Zhao et al., 2019) and metabolomic (Wang et al., 2015) studies have demonstrated that the chemical constituents of *Paeonia* plants from different habitats and varieties have certain differences; for example, researchers found that *P. lactiflora*, and *Paeonia. veitchii*. have nine common chromatographic peaks and four different chromatographic peaks, as evidenced by fingerprint comparisons and the fact that the phenolic acid contents are significantly different. For instance *P. veitchii.* contains more gallic acid derivatives [gallic acid, galloyl glucoses, methyl gallate, and galloylpaeoniflorin (GPF)] than the roots of *P. lactiflora.* (Xu et al., 2009). It was suggested, however, that *P. lactiflora.* and *P. veitchii*. are the original plants of Paeoniae Radix Rubra, although their chemical compositions were significantly different, such that these differences may affect their pharmacology and the pharmacological properties. The ancient Chinese medical book *Materia Medica Yan Yi* indicated that many varieties of *P. lactiflora.* existed, among which those growing in the mountains with red flowers and single leaves had better medicinal effects. This indicated that differences in the variety may be related to the pharmacology and the medicinal properties of the varieties *P. lactiflora.* Moreover, studies have shown that there are some differences in the medicinal properties, bioavailability and chemical composition of Paeoniae Radix Rubra derived from two original plants. For example, Paeoniae Radix Rubra has stronger anti-inflammatory, antiviral and antioxidant effects compared with *P. lactiflora.* (Parker et al., 2016). Therefore, it is of great significance to be able to study the differences in the chemical components of different varieties of *P. lactiflora.* as a means to promote the medicinal development of intersubgeneric hybrids of *P. lactiflora.*

In this study, we conducted a DUS evaluation of the consistency of nine intersubgeneric hybrid varieties of *P. lactiflora.*, in order to ensure the genetic stability of the selected varieties, and to guarantee the consistency and reproducibility of the analytical data on the chemical composition. High performance liquid chromatography (HPLC) was used to determine the PA content and establish the fingerprint profiles of the selected varieties. The fingerprint data were analyzed by cluster analysis (HCA), principal component analysis (PCA) and orthogonal partial least squares discriminant analysis (OPLS-DA). The chemical compositional analysis of the intersubgeneric hybrids of *P. lactiflora.* provide a theoretical basis and reference for evaluating their medicinal value.

## 2 Materials and methods

### 2.1 Materials

Paeoniflorin reference material (PA purity: 98.0%, batch number: wkq22032903), galloylpaeoniflorin reference material (GPF purity: 98.0%, batch number:wkq22011208), *P. lactiflora.* reference medicine (batch number: ycwkq22083111), and *P. veitchii*. reference medicine (batch number: ycwkq22083009) were purchased from Weikeqi Biological Technology Co., Ltd. (Sichuan, China).

Plant samples were provided by *P. lactiflora,* Germplasm Resource Nursery of Harbin Academy of Agricultural Sciences (126^o^55′ E, 45^o^8′ N). The altitude of the nursery is 115–120 m, the annual average temperature is 4.2°C, the maximum seasonal temperature is 36.7°C, and the minimum seasonal temperature is −37.7°C. The annual frost free period is 140∼160 days, and the maximum snow depth is 41 cm. The average annual relative humidity is about 66%, the average annual precipitation is 524.5 mm, and the average annual evaporation is 1,586.8 mm. The plant spacing in the growth plots was 50 cm × 50 cm and nine varieties of 5-year-old intersubgeneric hybrids of *P. lactiflora.* plants were selected. The sample variety names selected in this study include: “Diana”, “Adolescent Boy”, “Karl”, “Cameo”, “Mo Zi Ling”, “Qiao Ling”, “L- Bowl of Beauty”, “Zhong Sheng Fen”, “Zhu Guang”.

Two medicinal varieties of *P. lactiflora* were selected as reference medicinal varieties, they are the reference medicinal varieties “Zi Hai” and the wild medicinal *P. lactiflora.* “Zi Hai” was provided by *P. lactiflora* Germplasm Resource Nursery of Harbin Academy of Agricultural Sciences. The wild medicinal *P. lactiflora* plants were provided by the Medicinal Botanical Garden of Heilongjiang University of Chinese Medicine. Ma Wei, associate researcher of Heilongjiang University of Traditional Chinese Medicine, identified the two varieties of sample was *P. lactiflora.*

### 2.2 Methods

#### 2.2.1 DUS evaluation

The DUS (Distinctness, Uniformity, and Stability) evaluation of *P. lactiflora.* was based on the Agricultural Industry Standard of the People’s Republic of China (NY/T2225-2012—Guidelines for Specificity, Consistency and Stability Testing of New Plant Varieties (*P. lactiflora*.). The individual plants were measured in an independent growth cycle. The data indicators included: plant height, crown width, flower stems, flower branches and plant branches. The appearance characteristics of the plant included: the plant growth morphology, compound leaves, leaflets, flowers, buds, and other organs. Ten plants were selected randomly from the population of each variety and having a relatively consistent growth; each plant was observed and measured 3 times. Determination of the consistency requires that when the population is greater than 6, only one heterotypic plant is allowed. If a variety has consistency, it is concluded that the variety is stable.

#### 2.2.2 Determination of paeoniflorin content

##### 2.2.2.1 Chromatographic conditions

The experiment used Ultrafast high performance liquid chromatograph (HPLC, American Thermo Fisher Scientific, Ultimate 3000) and, the equipment is equipped with Chromeleon^®^ chromatographic data system. Chromatographic column: Diamonsil C18 (2) (250 mm × 4.6 mm, 5.0 μm); acetonitrile (A)-water plus 0.1% phosphoric acid (B) served as the mobile phase (14:86); the detection wavelength was 230 nm; the flow rate was 1.0 mL/min; the column temperature was 35°C.

##### 2.2.2.2 Preparation of test and reference solutions

The roots of different varieties of *P. lactiflora.* were taken, five duplicate samples wereset for each variety, ground and sieved and then air dried prior to determination of the content of PA, each sample shall be tested for 3 times.

###### 2.2.2.2.1 Test solution

The sample powder weighed using the electronic analytical balance (one in 10,000, Germany Mettler Toledo) was placed in a conical flask (50 mL) with stopper and 25 mL of methanol were added. After soaking for 4 h, ultrasound assisted solvent extraction 20 min (UASE, KQ-500DB, Kunshan, China) was carried out using ultrasound bath at 60°C, power 150 w, frequency 20 kHz. Aliquots of methanol were used to make up for the mass of solvent lost through evaporation. The test solution was centrifuged (220R centrifuge, Germany Hettich) and obtained by filtering the prepared solution through a 0.45 µm membrane filter.

The sample masses of the root powder for each variety were: “Diana” 0.42 g, “Adolescent Boy” 0.22 g, “Zhu Guang” 0.17 g, “Cameo” 0.16 g, “Mo Zi Ling” 0.14 g, “Qiao Ling” 0.24 g, “L—Bowl of Beauty” 0.16 g, “Zhong Sheng Fen” 0.15 g, “Karl” 0.13 g, control medicinal varieties “Zi Hai” 0.4 g, and wild medicinal *P. lactiflora.* artificially transplantedare variety 0.25 g.

###### 2.2.2.2.2 Reference solution

An appropriate amount of PA reference material was weighed accurately, and placed in a weighing bottle to prepare a reference solution of concentration 1 mg/mL. This solution was diluted for use according to the intended experimental requirements.

##### 2.2.2.3 Methodological investigations

###### 2.2.2.3.1 Standard curve

Reference solutions of concentration 1, 0.8, 0.6, 0.4, 0.2, and 0.05 mg/mL were prepared by serial dilution. In accordance with the chromatographic conditions described in Section 2.2.2.1, the area of the chromatographic peak corresponding to each concentration (3 repeat injections) was determined from the standard curve (the ordinate was the peak area; the abscissa was the concentration).

###### 2.2.2.3.2 Precision test

The intra day and inter day precision were measured, respectively. The reference solutions of concentrations 0.25, 0.5, and 0.75 mg/mL were taken, and six replicate injections into the HPLC system at each concentration were performed. The intra day precision was measured over 5 days, and the RSD value was calculated.

###### 2.2.2.3.3 Stability test

Fresh solutions corresponding to the same test solution and reference solution were injected into the HPLC system at 0, 2, 4, 8, 12, and 24 h, respectively, for quantitation, and the RSD values corresponding to the respective peak areas were calculated.

###### 2.2.2.3.4 Repeatability test

A known quantity of root powder of the same variety was spiked into each of the six test solutions, and each sample was injected into the HPLC system to calculate the RSD values for the corresponding peak area responses.

###### 2.2.2.3.5 Sampling recovery test

The six test sample solutions and the 0.5 mg/mL reference solutions were mixed 1:1 to calculate the recovery rate for the reference solution.

#### 2.2.3 Comparison of fingerprints for the different varieties of *P. lactiflora*

##### 2.2.3.1 Chromatographic conditions

The chromatographic conditions adopted were based on previously published work (Zaiyou et al., 2013; Chang et al., 2015). The chromatographic column: Diamonsil C18 (2) (250 mm × 4.6 mm, 5.0 μm); acetonitrile (A)-water plus 0.1% phosphoric acid (B) served as the mobile phase; degree of gradient elution 0 min ∼ 4 min, 95% ∼ 95% B; 4 min ∼ 7 min, 95% ∼ 90% B; 7 min ∼ 14 min, 90%–85% B; 14 min ∼ 22 min, 85% ∼ 80% B; 22 min ∼ 34 min, 80% ∼ 80% B; 34 min ∼ 35 min, 80% ∼ 78% B; 35 min ∼ 45 min, 78% ∼ 75% B; 45 min ∼ 50 min, 75% ∼ 95% B; flow rate 0.8 mL/min; detection wavelength 220 nm; column temperature 35°C.

##### 2.2.3.2 Preparation of test solution and reference solution

Test solution: Root powder of five duplicate samples of each variety of *P. lactiflora.* (0.1 g) was placed in a conical flask (50 mL) with stopper and 5 mL of methanol were added. The samples were prepared according to Section 2.2.2.2.

Reference solution: Samples of *P. veitchii*. an*d P. lactiflora.* Reference medicine substances (0.1 g) were weighed accurately, and used to prepare reference solutions according to the method in Section 2.2.2.2.

##### 2.2.3.3 Methodological investigation

###### 2.2.3.3.1 Precision test

The solution of *P. lactiflora.* reference medicine material served as the reference sample, and a sample of dried root powder was taken to prepare the test substance. According to the chromatographic conditions described in Section 2.2.3.1, 6 consecutive injections into the HPLC system were made, and the relative peak areas and relative retention times for each chromatographic peak were measured with peak No. 4 (Figures 4A, B) serving as the S peak (Main reference peak).

###### 2.2.3.3.2 Repeatability test

The solution of *P. lactiflora.* Reference medicine material served as the sample, and samples of dried root powder were taken to prepare six test solutions to be analyzed in parallel and tested according to the chromatographic conditions described in Section 2.2.3.1. The peak areas and retention times for each common peak were measured and as before peak No. 4 served as the S peak.

###### 2.2.3.3.3 Stability test

The freshly prepared *P. lactiflora.* reference medicine test solution was injected into the HPLC system at 0, 2, 4, 6, 8, 10, 12, and 24 h, respectively, in accordance with the chromatographic conditions specified in Section 2.2.3.1. The peak areas and retention times for each chromatographic peak were determined with peak No. 4 serving as the S peak.

##### 2.2.3.4 Data analysis

The unsupervised pattern recognition techniques, cluster analysis (HCA) and principal component analysis (PCA), as well as the supervised pattern recognition technique orthogonal partial least squares discriminant analysis (OPLS-DA) were used to analyze the chromatographic data. The analysis software were IBM SPSS Statistics 19 (IBM Corp., Armonk, NY, United States) and Simca 14.1(Umetrics, Malmo, Sweden).

## 3 Results

### 3.1 Morphological characterization

**FIGURE 1:**
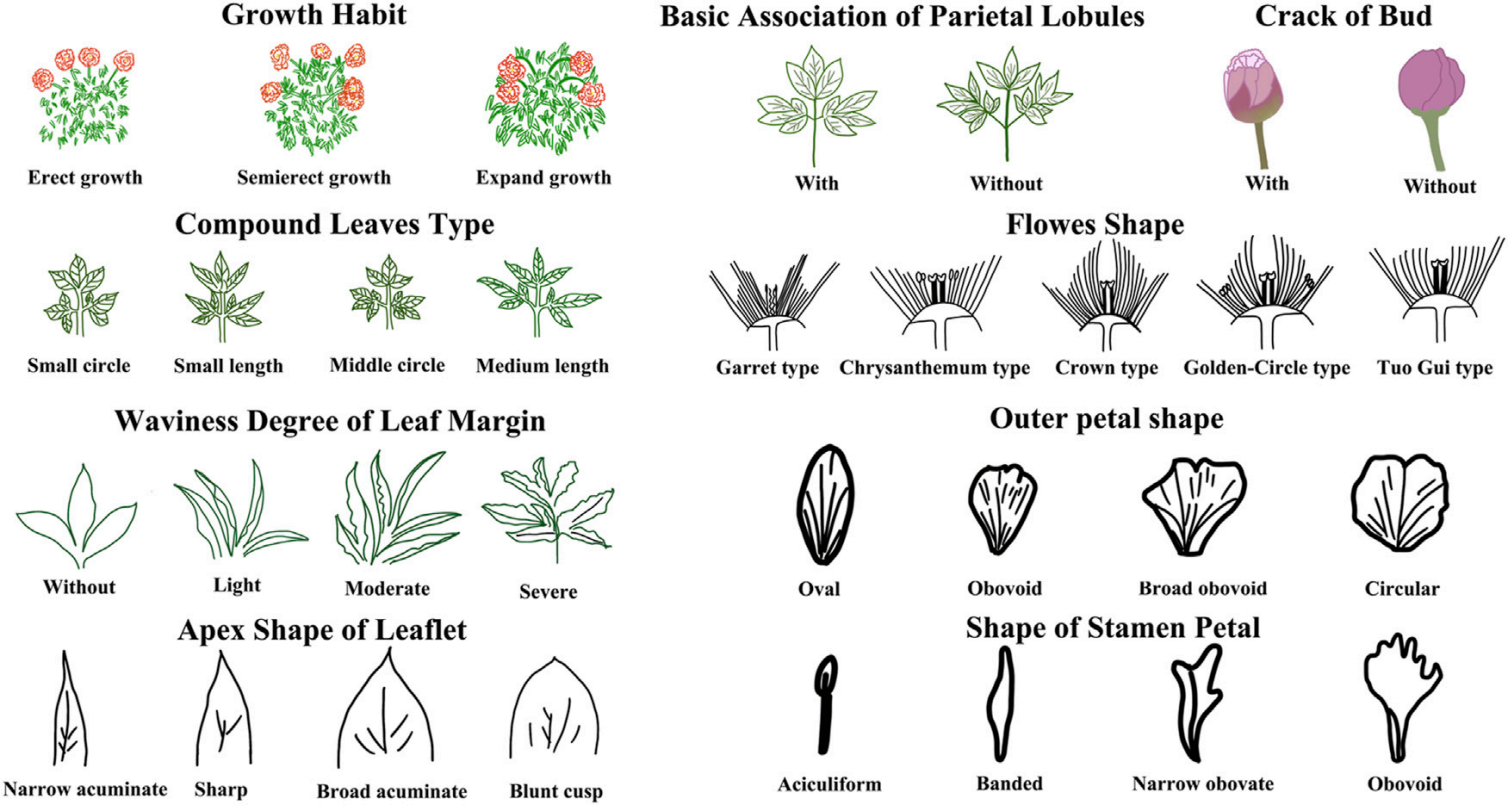
DUS morphological feature analysis and the standard atlas for evaluation.

**FIGURE 2:**
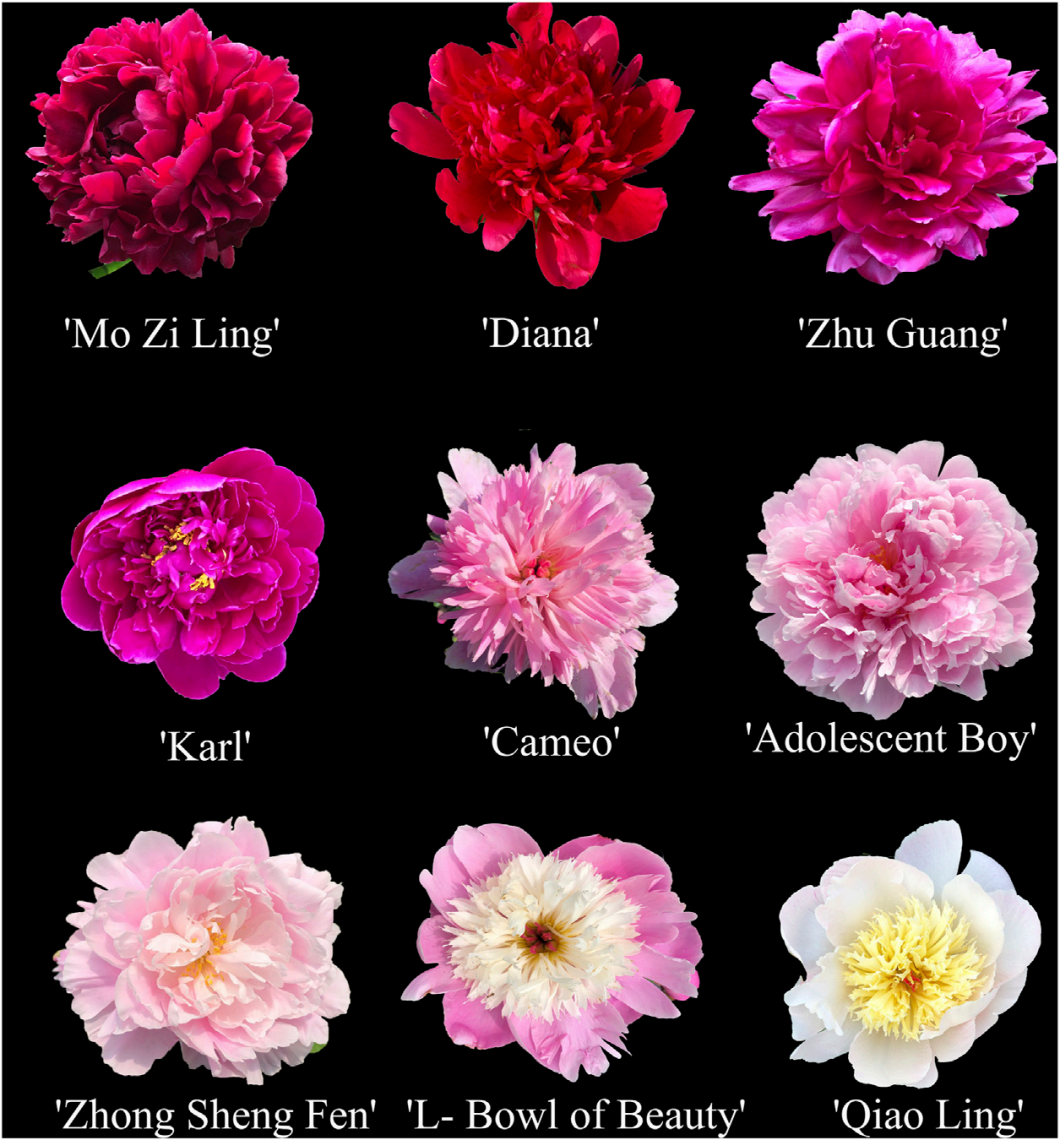
Samples of the nine intersubgeneric hybrids of *P. lactiflflora* used in this study.

The evaluation results for DUS confirmed that the nine varieties of intersubgeneric hybrids of *P. lactiflora.* plants exhibited consistency and stability among the different individuals of the same variety, and there was specificity between the different varieties; an atlas of the characteristic evaluation standards is presented in Figure 1. We investigated the growth characteristics of nine varieties of the intersubgeneric hybrids of *P. lactiflora.* From four aspects: Morphological characteristics of the plants (Tables 1, 2), the leaves (Table 3), the flowers (Table 4) and the reproductive organs (Table 5) and which were gauged with respect to the DUS evaluation criteria. The appearance of the nine varieties of the flowers is shown in Figure 2, the varieties being arranged according to the depth of color. In the case of the plant samples selected, except for “Mo Zi Ling”, which was a monochrome flower, the other varieties were of a composite color.

**TABLE 1:** Morphological characteristics of the plants.

Code	Cultivar	Color	Number of colors	Variety source	Growth habit	Number of flowers
1	“Mo Zi Ling”	Dark purple	1	China	Expand growth	Middle
2	“Qiao Ling”	White	3	China	Erect growth	Middle
3	“Zhong Sheng Fen”	Blue Pink	2	China	Semierect growth	Few
4	“Zhu Guang”	Purplish red	2	China	Semierect growth	Few
5	“Adolescent Boy”	Rose hermosa	3	Holland	Semierect growth	Middle
6	“L- Bowl of Beauty”	Composite color	3	Holland	Erect growth	Middle
7	“Karl”	Purplish red	2	Holland	Erect growth	Middle
8	“Cameo”	Peach	2	Holland	Erect growth	Middle
9	“Diana”	Red	2	Holland	Expand growth	Few

“Number of colors” refers to the number of colors in each flower. The color of the flower is based on the main color. In case of any discrepancy as a result of visual inspection, the indicated data of “The Royal Horticultural Society’s color scale” (RHSCC) shall prevail.

**TABLE 2:** Growth parameters of the plants.

Code	Cultivar	Character parameters (cm)					
		Plant height	Crown diameter	Corolla diameter	Flower branch length	Flower branchdiameter	Number of branches
1	“Mo Zi Ling”	75.10 ± 4.89	87.70 ± 5.83	12.48 ± 0.28	63.50 ± 3.93	0.76 ± 0.07	11.30 ± 1.23
2	“Qiao Ling”	50.30 ± 3.98	65.40 ± 5.70	16.95 ± 0.40	39.40 ± 6.23	0.54 ± 0.05	10.80 ± 0.98
3	“Zhong Sheng Fen”	51.75 ± 4.94	64.50 ± 6.36	11.88 ± 0.44	39.40 ± 6.60	0.53 ± 0.06	9.70 ± 0.90
4	“Zhu Guang”	50.60 ± 3.95	64.50 ± 4.65	13.36 ± 0.40	45.50 ± 5.18	0.61 ± 0.07	10.60 ± 1.11
5	“Adolescent Boy”	60.40 ± 3.32	64.70 ± 5.80	15.36 ± 0.34	51.60 ± 4.27	0.69 ± 0.07	7.00 ± 0.77
6	“L- Bowl of Beauty”	62.40 ± 5.08	73.00 ± 7.14	16.49 ± 0.63	49.90 ± 5.20	0.68 ± 0.07	8.20 ± 1.08
7	“Karl”	50.20 ± 4.96	56.60 ± 3.17	15.74 ± 0.31	39.30 ± 3.32	0.56 ± 0.05	7.90 ± 0.83
8	“Cameo”	51.10 ± 3.45	65.30 ± 3.20	12.74 ± 0.26	41.00 ± 6.08	0.61 ± 0.05	5.60 ± 0.49
9	“Diana”	49.90 ± 4.61	82.10 ± 7.30	13.57 ± 0.35	41.70 ± 5.00	0.76 ± 0.07	5.90 ± 0.83

**TABLE 3:** Morphological characteristics of the leaves.

Code	Compound leaves						Leaflet			
	Cultivar	Number of secondary leaflets of terminal leaflet	Type	Angle of compound leaf petiole and flower branch	Upper surface color	Petiole color	Degree of involution	Waviness degree of leaf margin	Apex shape	Basic association of parietal lobules
1	“Mo Zi Ling”	3	Medium length	Medium	Green	Purplish red	Severe	Moderate	Sharp	Without
2	“Qiao Ling”	3	Medium length	Small	Dark green	Purplish red	Severe	Light	Broad acuminate	Without
3	“Zhong Sheng Fen”	3 or 4	Middle circle	Medium	Dark green	Purplish red	Moderate	Light	Sharp	With or without
4	“Zhu Guang”	3	Medium length	Small	Dark green	Purplish red	Severe	Light	Sharp	Without
5	“Adolescent Boy”	3	Small length or medium length	Large	Green	Purplish red	Moderate	Light	Narrow acuminate	Without
6	“L-Bowl of Beauty”	3	Medium length	Medium	Green	Purplish red	Moderate	Moderate	Sharp	Without
7	“Karl”	3	Small circle	Medium	Green	Purplish red	Moderate	Moderate	Sharp	Without
8	“Cameo”	3	Small length	Large	Dark green	Purplish red	Light	Light	Sharp	Without
9	“Diana”	3–5	Middle circle	Large	Dark green	Green	Light	Without	Blunt cusp	With

Angle of compound leaf petiole and flower branch: “small” within 30°, “medium” around 45°, and “large” around 60°.

**TABLE 4:** Morphological characteristics of the flowers.

Code	Cultivar	Flower posture	Shape	Sepal petalization	Pedicel color	Outer petal shape	Outer petal hardness
1	“Mo Zi Ling”	Oblique upward	Garret type	With	Purplish red	Obovoid	Moderate
2	“Qiao Ling”	Erect	Tuo Gui type	Without	Purplish red	Obovoid	Hard
3	“Zhong Sheng Fen”	Nodding	Crown type	Without	Greenish reddish	Obovoid	Soft
4	“Zhu Guang”	Oblique upward	Crown type	With	Purplish red	Oval	Soft
5	“Adolescent Boy”	Oblique upward	Chrysanthemum type	With	Green	Obovoid	Moderate
6	“L-Bowl of Beauty”	Oblique upward	Tuo GUI type	With	Purplish red	Obovoid	Moderate
7	“Karl”	Erect	Golden-Circle type	With	Purplish red	Circular	Moderate
8	“Cameo”	Erect	Tuo GUI type	With	Green	Obovoid	Hard
9	“Diana”	Oblique upward	Tuo GUI type	With	Green	Circular	Hard

**TABLE 5:** Morphological characteristics of the reproductive organs.

Code	Cultivar	Stamen			Stamen petal			Pistil		Pistil petal	Bud		
		Degree of petalization	Anther residue	Filament color	Shape	Color relative to outer petal	Color	Degree of petalization	Stigma color	Color	Crack	Color	Whether there is lateral bud
1	“Mo Zi Ling”	Rare	Without	Yellow	Obovoid	Same	Dark purple	Without	Without	Without	With	Purplish red	With
2	“Qiao Ling”	Whole	Without	Without	Banded	Same	White	Without	Purplish red	Without	Without	White	Without
3	“Zhong Sheng Fen”	Rare	Without	Yellow	Obovoid	Same	Blue Pink	Without	Without	Without	With	Purplish red	Without
4	“Zhu Guang”	Part	Without	Yellow	Obovoid	Same	Purplish red	Without	Without	Without	Without	Purplish red	Without
5	“Adolescent Boy”	Part	Without	Yellow	Mixed form	Same	Rose hermosa	Without	Without	Without	Without	Rose hermosa	Without
6	“L-Bowl of Beauty”	Whole	Without	Without	Narrow obovate	Different	White	Without	Purplish red	Without	Without	Purplish red	With
7	“Karl”	Rare	Without	Yellow	Obovoid	Same	Purplish red	Without	Without	Without	Without	Purplish red	Without
8	“Cameo”	Whole	Without	Without	Narrow obovate	Same	Purplish red	Without	Purplish red	Without	Without	Purplish red	Without
9	“Diana”	Whole	Without	Without	Obovoid	Same	Red	Without	Green	Without	Without	Purplish red	Without

### 3.2 Results of methodological investigations

#### 3.2.1 Standard curve

The average retention time of PA was 12.101 min (*n* = 6), and the equation for the standard curve of PA was *Y = 63.743X–0.7085* (*R* ^2^ = 0.999), and the linear range was 0.05 mg/mL∼1.00 mg/mL. The limit of detection (LOD) calculated with the signal to noise ratio (S/N) = 3 was 0.32 μg/mL, the limit of quantification (LOQ) calculated with the signal to noise ratio (S/N) = 10 was 1.09 μg/mL.

#### 3.2.2 Precision test

The RSD value for the intraday precision was 0.423%, and that for the inter day precision was 0.588%. Given that both precision values were less than 2.0%, the precision for instrumental measurement was considered good.

#### 3.2.3 Stability test

The results for the stability study indicated that the RSD values for the peak areas for the test solution and the reference solution within 24 h were 1.24% and 0.895%, respectively, which indicated that the stability of the two types of solution were good within a 24 h period.

#### 3.2.4 Repeatability test

The RSD value for the repeatability experiment was 0.368%, indicating that the repeatability of the detection method was good.

#### 3.2.4 Sampling recovery test

The results for sample recovery test showed that the range for the recovery rate for the reference materials was 98.040%∼100.333%, and the RSD value was 0.973%, which was less than 2.0%, thus conforming to the requirements for DUS evaluation.

### 3.3 Analytical values for the content of PA

**FIGURE 3:**
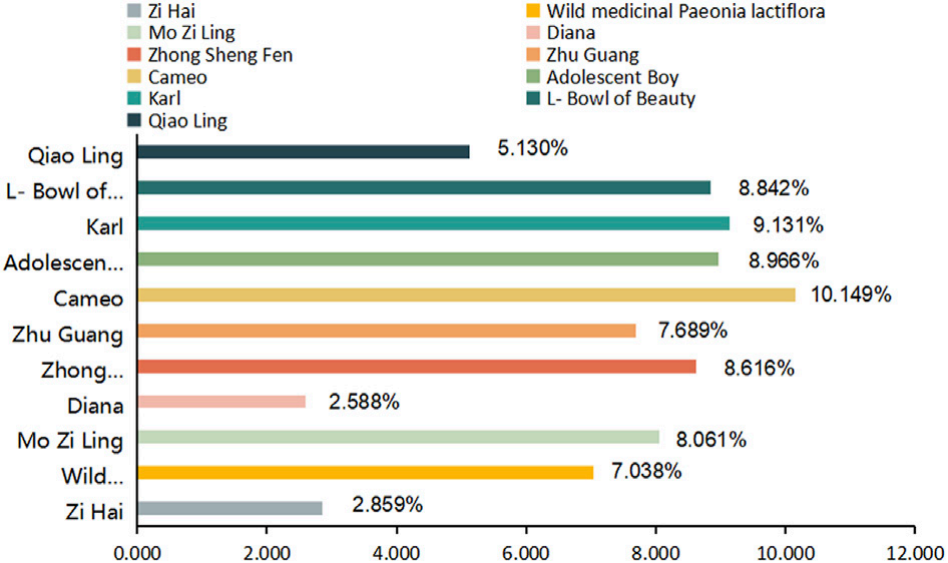
Content of paeoniflorin (PA) in the nine intersubgeneric hybrids of *P. lactiflora.*

The analytical values for the content of PA in the roots of the 11 varieties of *P. lactiflora.* are shown in Figure 3 IBM SPSS Statistics 19 software was used for statistical analysis, there was no significant difference in the content of PA in the same variety group (*p* > 0.05), and there was significant difference between the intersubgeneric hybrids varieties compared with the two medicinal varieties (*p* < 0.01). According to the requirements of the Pharmacopoeia of the People’s Republic of China **(** Chinese Pharmacopoeia Commission, 2020), the PA content of Radix Paeoniae Rubra should exceed 1.8%. The PA contents in the roots of the reference medicinal varieties “Zi Hai” and wild medicinal *P. lactiflora.* were 2.859% and 7.038%, respectively. The order for the PA content in the roots of the intersubgeneric hybrids of *P. lactiflora.* was as follows: “Cameo” > “Karl” > “Adolescent Boy” > “L-Bowl of Beauty” > “Zhong Sheng Fen” > “Mo Zi Ling” > “Zhu Guang” > wild medicinal *P. lactiflora.* > “Qiao Ling” > “Zi Hai” > “Diana”. The PA contents in the roots of these varieties were higher than the standard specified in the Ch.P for Radix Paeoniae Rubra; also, the “Cameo” variety had the highest content, reaching 10.149%. In addition, there were seven varieties whose PA content in the roots exceeded that of wild medicinal *P. lactiflora.*, and eight varieties whose PA content exceeded that of reference medicinal variety “Zi Hai”. These results demonstrated clearly that the intersubgeneric hybrids of *P. lactiflora.* can be used as an important reserve resource for extraction of PA. However, whether the roots of these *P. lactiflora.* varieties can be used as an alternative to the original Chinese medicinal plant Radix Paeoniae Rubra needs to be further studied.

### 3.4 Fingerprint analyses of different varieties of *P. lactiflora*

#### 3.4.1 Precision test

The results for the precision test indicated that the RSDs for the relative retention times and relative peak areas for each chromatographic peak in the test solution of *P. lactiflora.* were less than 5%, which indicated that the precision of the method was good.

#### 3.4.2 Repeatability test

The results of repeatability test showed that the RSDs for the relative retention times and the relative peak areas for each chromatographic peak in the test solution of *P. lactiflora.* were less than 5%, which indicated that the repeatability of the method was good.

#### 3.4.3 Stability test

The stability test results for a 24 h period indicated that the RSDs for the relative retention times and relative peak areas for each chromatographic peak in the test solution of *P. lactiflora.* were less than 5%, which indicated that the stability of the test solution was acceptable for the 24 h period investigated.

#### 3.4.4 Similarity evaluation

**FIGURE 4:**
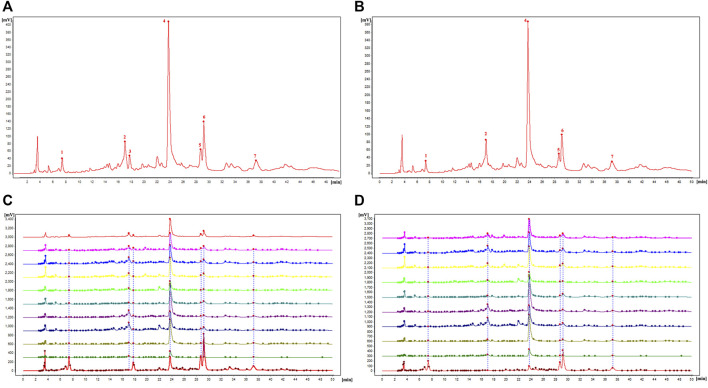
Characteristic map and fingerprint analysis of the nine intersubgeneric hybrids of *P. lactiflora* referred to reference medicine of *P. lactiflora.* and *P. veitchii.* [**(A)** Identification of chromatographic peaks with *P. lactiflora.* as reference; **(B)** Identification of chromatographic peaks with *P. veitchii.* as reference; **(C)** Characteristic map and fingerprint analysis of nine intersubgeneric hybrids of *P. lactiflora* and reference medicine of *P. lactiflora.*; **(D)** Characteristic map and fingerprint analysis of nine intersubgeneric hybrids of *P. lactiflora* and reference medicine of *P. veitchii.*].

The similarity evaluation software for analysis of the chromatographic fingerprint profiles of TCM was used to calculate the similarity of the fingerprints for the two reference medicinal materials and the nine sample varieties. The characteristic response profiles and fingerprint overlays are presented in Figure 4, identification of chromatographic peaks are presented in Figures 4A, B, and Characteristic maps are presented in Figures 4C, D. The similarity values for the fingerprints of the nine sample varieties compared with *P. lactiflora.* and *P. veitchii*. are shown in Table 6 and Table 7, respectively. The respective peak area and retention time data are listed in Table 8.

**TABLE 6:** Similarity evaluation based on the chromatography of *P. lactiflflora* as reference.

Sample	S1	S2	S3	S4	S5	S6	S7	S8	S9	S10	R
S1	1.000	0.456	0.415	0.485	0.410	0.547	0.474	0.524	0.595	0.631	0.639
S2	0.456	1.000	0.973	0.989	0.991	0.972	0.996	0.989	0.970	0.964	0.973
S3	0.415	0.973	1.000	0.935	0.989	0.988	0.967	0.981	0.915	0.923	0.950
S4	0.485	0.989	0.935	1.000	0.971	0.943	0.993	0.977	0.982	0.970	0.970
S5	0.410	0.991	0.989	0.971	1.000	0.979	0.990	0.991	0.949	0.949	0.962
S6	0.547	0.972	0.988	0.943	0.979	1.000	0.969	0.990	0.943	0.957	0.981
S7	0.474	0.996	0.967	0.993	0.990	0.969	1.000	0.992	0.975	0.970	0.978
S8	0.524	0.989	0.981	0.977	0.991	0.990	0.992	1.000	0.968	0.973	0.988
S9	0.595	0.970	0.915	0.982	0.949	0.943	0.975	0.968	1.000	0.996	0.984
S10	0.631	0.964	0.923	0.970	0.949	0.957	0.970	0.973	0.996	1.000	0.991
R	0.639	0.973	0.950	0.970	0.962	0.981	0.978	0.988	0.984	0.991	1.000

S1, *P. lactiflora.*; S2, ”Diana”; S3, “Adolescent Boy”; S4, “Karl”; S5, “Cameo”; S6, “Mo Zi Ling”; S7, “Qiao Ling”; S8, “L-Bowl of beauty”; S9, “Zhong Sheng Fen”; S10, “Zhu Guang”.

**TABLE 7:** Similarity evaluation based on the chromatography with *P. veitchii.* as reference.

Sample	S1′	S2	S3	S4	S5	S6	S7	S8	S9	S10	R
S1′	1.000	0.335	0.296	0.375	0.290	0.433	0.364	0.413	0.483	0.520	0.471
S2	0.335	1.000	0.974	0.989	0.992	0.972	0.997	0.990	0.970	0.965	0.986
S3	0.296	0.974	1.000	0.935	0.989	0.989	0.967	0.981	0.917	0.926	0.966
S4	0.375	0.989	0.935	1.000	0.971	0.943	0.993	0.977	0.983	0.971	0.980
S5	0.290	0.992	0.989	0.971	1.000	0.979	0.990	0.991	0.951	0.951	0.979
S6	0.433	0.972	0.989	0.943	0.979	1.000	0.970	0.991	0.944	0.957	0.985
S7	0.364	0.997	0.967	0.993	0.990	0.970	1.000	0.992	0.978	0.973	0.990
S8	0.413	0.990	0.981	0.977	0.991	0.991	0.992	1.000	0.970	0.975	0.996
S9	0.483	0.970	0.917	0.983	0.951	0.944	0.978	0.970	1.000	0.996	0.985
S10	0.520	0.965	0.926	0.971	0.951	0.957	0.973	0.975	0.996	1.000	0.989
R	0.471	0.986	0.966	0.980	0.979	0.985	0.990	0.996	0.985	0.989	1.000

S1′, *Paeonia veitchii Lync*h. The varieties represented by S2–S10 are the same as those in Table 6.

**TABLE 8:** Chromatographic peak areas for *P. lactiflora.* and *P. veitchii.* as reference.

Serial number	Retention time	S1	S1′	S2	S3	S4	S5	S6	S7	S8	S9	S10	Control fingerprint
1	7.381	80.565	43.965	1.589	5.739	2.403	2.153	6.896	4.629	5.464	4.953	5.640	12.003
2	17.039	6.327	1.638	16.483	12.731	91.308	58.880	11.261	28.069	35.709	70.539	50.294	38.160
3	17.767	63.926	—	4.693	6.951	12.994	12.721	11.291	3.144	8.020	22.708	20.989	16.744
4	23.737	108.472	32.342	55.954	209.955	212.285	308.78	168.445	90.699	186.562	177.397	162.495	168.104
5	28.712	111.781	59.733	3.090	8.223	23.079	12.702	16.030	7.301	21.130	5.187	5.893	21.442
6	29.182	278.352	137.959	6.402	13.313	38.303	25.211	34.577	13.579	36.524	65.623	66.415	57.830
7	37.234	75.184	37.144	7.853	25.576	20.129	17.408	26.127	7.432	16.903	17.217	15.069	22.890

A total of seven chromatographic peaks were identified with reference to the standard medicinal material *P. lactiflora.*, and six chromatographic peaks were identified with reference to the standard medicinal material *P. veitchii*.; in the latter case, peak No. 3 with a retention time of 17.767 min was not observed in *P. veitchii*. Identification of the characteristic peaks by the method of comparison with reference substance which revealed peak No. 4 corresponded to PA, and No.6 corresponded to GPF. The results of the similarity comparison showed that the nine varieties of *P. lactiflora.* had high similarity with each other, but low similarity with the reference varieties *P. lactiflora.* and *P. veitchii*. This finding indicated that there was large differences in the peak areas between intersubgeneric hybrids and the medicinal varieties, which may be related to hybridization and domestication of the sample varieties.

### 3.5 Stoichiometric analysis

The stoichiometric analysis is divided into supervised and unsupervised models, in which HCA and PCA are unsupervised models, which are often used to observe the classification trend of samples. OPLS-DA is supervised mode, which shows which variables cause the difference of samples.

#### 3.5.1 Cluster analysis (HCA)

**FIGURE 5:**
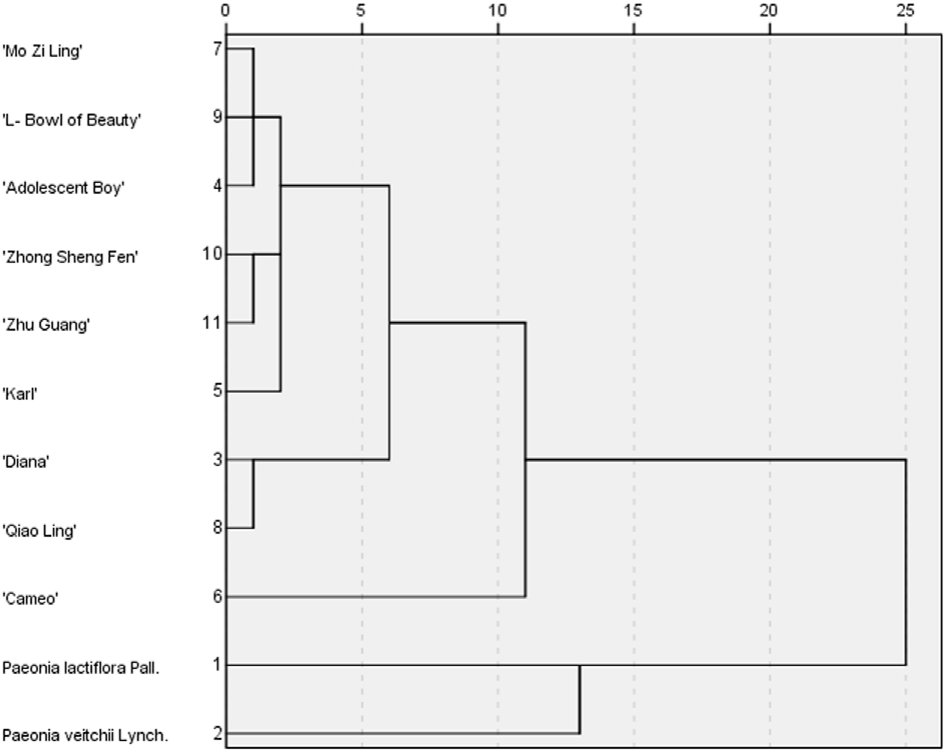
Cluster analysis diagram.

The peak area values for the chromatographic peaks of the nine varieties of intersubgeneric hybrids of *P. lactiflora.* and the two medicinal reference varieties *P. lactiflora.* and *P. veitchii*. Served as the variables, and the *IBM SPSS Statistics 19* software was used for HCA. After the peak area values were standardized, the distance between samples was represented by the *Euclidean* distance squared, and HCA was carried out through inter group connection (Figure 5). The results showed that when the distance between groups was 15, the samples were divided into two categories: nine varieties of intersubgeneric hybrids of *P. lactiflora.* clustered into one branch, and the two kinds of medicinal reference herbs clustered into one branch. When the distance between groups was 10, the samples could be classified into four categories: They were *P. lactiflora.*, *P. veitchii*, and “Cameo” in each was a category, and the other eight intersubgeneric hybrids of *P. lactiflora.* belonged to one category. The HCA results were basically consistent with the fingerprint similarity evaluation results.

#### 3.5.2 Principal component analysis (PCA)

**FIGURE 6:**
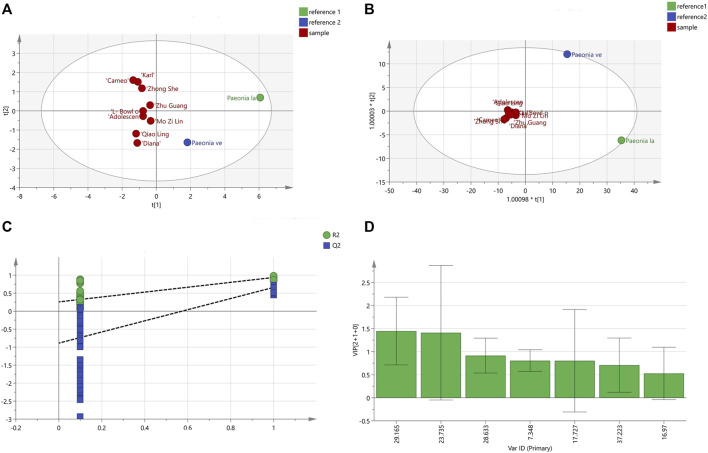
PCA scores diagram **(A)** and OPDS-DA analysis results; **(B)** OPDS-DA scores diagram; **(C)** Model permutation test diagram; **(D)** VIP diagram.

The peak area values for the chromatography were imported into the Simca 14.1 software for PCA analysis, and five principal components were generated, and where the independent variable fitting index (R^2^X) = 0.997 and the model prediction index (Q^2^) = 0.752 were all greater than 0.5, indicating that the model fitting results were valid. The contribution rate of principal component 1 was 68.3%, the contribution rate of principal component 2 was 20.2%, the contribution rate of principal component 3 was 7.12%, the contribution rate of principal component 4 was 3.26%, and the contribution rate of principal component 5 was 0.6%. The cumulative contribution rate of principal components 1 and 2 was more than 85%. The PCA scores for the 11 varieties of *P. lactiflora.* are displayed in Figure 6A. The results showed that *P. lactiflora.* was distributed on the positive half axis, while *P. veitchii*. was distributed on the negative half axis, and nine intersubgeneric hybrids of *P. lactiflora.* were relatively concentrated. In addition, in the score chart, “Carl”, “Cameo” and “Zhong Sheng Fen” were closer to each other; “Zhu Guang”, “Adolescent Boy”, “L- Bowl of Beauty” and “Mo Zi Ling” were closer to each other; and “Qiao Ling” and “Diana” were closer to each other. It is suggested that although the nine intersubgeneric hybrids of *P. lactiflora.* were clustered into one branch in HCA analysis, in fact, there were still some differences among the components of the different varieties.

#### 3.5.3 Orthogonal partial least squares discriminant analysis (OPLS-DA)

The peak area values for the samples were analyzed by OPLS-DA, and the results are shown in Figure 6B. The scores of the two medicinal reference varieties *P. lactiflora.* and *P. veitchii*. and the nine intersubgeneric hybrids of *P. lactiflora* were clearly separated on the scatter plot. The R^2^X value was 0.93, the dependent variable fitting index (R^2^Y) was 0.908, and the Q^2^ value was 0.535, hence *R* ^2^ and Q^2^ both exceeded 0.5, indicating that the model fitting result was acceptable. The intercept values of *R* ^2^ and Q^2^ were 0.258 and −0.888, respectively. It can be concluded that by observing the test chart for the model permutation (Figure 6C), the models were randomly arranged some 200 times for replacement inspection, and the results showed that the intercept values of all the points on the left of *R* ^2^ and Q^2^ were lower than the rightmost points, which indicated that the established OPLS-DA model did not show over fitting.

The prediction result of OPLS-DA was similar to that of PCA. In general, the intersubgeneric hybrids of the *P. lactiflora* varieties were clearly clustered, which indicated that the chemical composition of the intersubgeneric hybrids of *P. lactiflora* varieties were distinctly different from that of the medicinal reference varieties. Simca 14.1 software was used to continue to generate the variable VIP diagram (Figure 6D) to predict the significance of the variables. The VIP values were ranked as S6 (GPF) >S4 (PA) > S5 > S1 > S3 > S7 > S2. The standard for screening the chromatographic peaks with significant differences was that for the VIP >1.0, and the two chromatographic peaks with VIP>1.0 were S6 (GPF) and S4 (PA). This indicates that the difference of varieties caused by hybridization and domestication will affect the content of PA, the main medicinal component.

## 4 Discussion

DUS is an intellectual property right system introduced by the International Union for the Protection of New Plant Varieties (UPOV) in 1961 for the determination and testing of new varieties (Yang et al., 2021). The DUS approach is also often used as an important basis and standard for the study of artificial selection and evaluation, and for the identification of the environmental impact on plant morphology (Wang L et al., 2022; Liu et al., 2019). For the study of the chemical components of plants of the same variety, the specificity, consistency and stability of the group should be determined first to ensure the universality and representativeness of the analytical results. Therefore, the DUS evaluation system was used to observe and measure the morphology of the population of the samples under investigation. The results showed that for the same living environment, the nine intersubgeneric hybrids of *P. lactiflora.* of the different varieties showed significant group differences and genetic diversity. This specificity was not only shown in terms of the color of the flower, but also in the plant morphology, the leaves, the petals, the reproductive organs and other parts. However, the population of the same varieties can maintain a high degree of stability and consistency, which ensures the reliability of data collection.

The root is the main medicinal part of *P. lactiflora.* In recent years, there have been many studies on the chemical composition of the root of *Paeonia* plants. In addition to analyzing the differences in the chemical composition of the different varieties, special attention has also been paid to the main active ingredient PA (Shi et al., 2016). PA is a key chemical component in the *Paeonia Sect. Paeonia*, which is a kind of monoterpene compound with “cage” pinane skeleton. At the same time, PA can also be used as a reference material for the single-marker (QAMS) method for quantitative analysis of the various components in the root of *P. lactiflora.* (Shen et al., 2021). Therefore, PA has important theoretical and practical significance with respect to clarifying the medicinal value of intersubgeneric hybrids of *P. lactiflora.* based on determining the differences in the content of PA in the different varieties of *P. lactiflora.* The analytical values for fingerprint analysis in this study showed that the content of PA in the nine intersubgeneric hybrids of *P. lactiflora.* were quite different, and the VIP variable map generated by the Simca 14.1 software showed that the chromatographic peaks S6 (GPF) and S4 (PA) were significant variables. Therefore, in addition to PA, the diversity of varieties will also affect the synthesis of GPF, GPF is also one of monoterpene glycosides, monoterpene glycosides are the main effective components of *P. lactiflora.*, which have rich biological activities and have significant effects on anti-inflammatory and anti-tumor (Zhang and Wei, 2020; Zhou et al., 2022).

With respect to HPLC, selection and optimization of the mobile phase was studied and it was found that the chromatography using acetonitrile as the organic phase was better than that of methanol in terms of signal quantitation and fingerprint analysis. Tailing of the main peak was aggravated when methanol served as the mobile phase. In addition, it was necessary to add 1% phosphoric acid to the aqueous phase to adjust the peak shape and tailing phenomenon. Finally, acetonitrile (A)-water plus 0.1% phosphoric acid (B) was selected as the mobile phase for determination and fingerprint analysis.

The results of this study further confirmed that there were significant differences in chemical composition and content among the nine intersubgeneric hybrids of the *P. lactiflora.* varieties compared with *P. lactiflora.* and *P. veitchii*., where the fingerprint similarity evaluation was low, the values all being less than 0.7. However, the analytical values showed that the nine intersubgeneric hybrids of *P. lactiflora.* were elevated in PA, compared with the wild and cultivated medicinal varieties, and only the content of ‘Diana’ was lower than that of the two medicinal varieties, which confirmed that the intersubgeneric hybrids of *P. lactiflora.* had the potential to serve as sources of raw material for extraction of PA.

The HCA and PCA analysis results for the chromatographic peak areas in the fingerprint analysis clearly distinguished the reference medicinal materials from the root extracts of the intersubgeneric hybrids of *P. lactiflora., P. lactiflora.* and *P. veitchii*. which were, respectively, distributed on the PCA distribution map, and they were also far away from the intersubgeneric hybrids of *P. lactiflora.,* while the nine kinds of intersubgeneric hybrids of *P. lactiflora.* were distributed more densely. Two differential marker substances S6 and S4 were found using OPLS-DA, wherein S4 was PA and S6 was GPF, indicating that the formation of PA and GPF was related to differences in the varieties.

## 5 Conclusion

The research results confirmed that the growth status of intersubgeneric hybrids of *P. lactiflora.* plants was stable, and the content of compounds in the same variety was also relatively stable, especially the monoterpene glycosides PA and GPF were the two chemical components that were greatly affected by the variety. Fingerprint similarity evaluation and stoichiometric analysis showed that the similarity between the intersubgeneric hybrids of *P. lactiflora.* and the original medicinal plants was low, but the experimental varieties we chose were closer to *P. lactiflora.* which compared with *P. veitchii*.

This study provides an important reference and basis for the determination of the medicinal value of the intersubgeneric hybrids of *P. lactiflora*, hence ongoing research on the development of medicinal varieties and resources of *P. lactiflora* is warranted.

## Data Availability

The original contributions of this study are included in the present article or in the supplementary materials. Inquiries concerning this work can be directed to the corresponding authors.
